# Feasibility, safety, and utility of bronchoscopy in patients with ARDS while in the prone position

**DOI:** 10.1186/s13054-018-1983-3

**Published:** 2018-03-02

**Authors:** Or Kalchiem-Dekel, Carl B. Shanholtz, Jean Jeudy, Ashutosh Sachdeva, Edward M. Pickering

**Affiliations:** 1 Division of Pulmonary, Critical Care, and Sleep Medicine, University of Maryland School of Medicine, 110 South Paca Street, Baltimore, MD 21201 USA; 2 Department of Diagnostic Radiology and Nuclear Medicine, University of Maryland School of Medicine, 110 South Paca Street, Baltimore, MD 21201 USA

## ᅟ

**Fig. 1:**
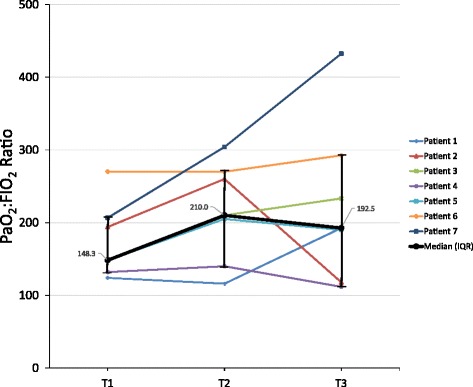
Evolution of PaO_2_ to FIO_2_ ratio from pre bronchoscopy (T1) to 24 h (T2) and 72 h (T3) post bronchoscopy (*n* = 7). IQR interquartile range, PaO_2_ partial pressure of arterial oxygen, FIO_2_ fractional concentration of inspired oxygen

Prone positioning (PP) was shown to reduce mortality in mechanically ventilated (MV) patients with severe ARDS [1]. Despite its common use, safety concerns inhibit use of flexible bronchoscopy (FB) in patients with ARDS, and there are few reports of FB performed in PP [2]. We reviewed all adults receiving FB in PP in one institution between April 2016 and September 2017. The study was approved by the institutional review board. Four men and three women were identified (Table 1). In five patients, FB was indicated for clearance of thick secretions, and in two patients for microbial analysis. The mode of mechanical ventilation was not changed for FB, but FIO_2_ was universally set to 100%. All subjects had invasive hemodynamic and pulse oximetry monitoring. End-tidal carbon dioxide (EtCO_2_) was monitored in 3/7 subjects. With the subject’s head tilted to the side, the bronchoscope was advanced into the airways, repeatedly, and in short cycles, allowing time for oxygenation, ventilation, and lung recruitment between insertions. Therapeutic aspiration was performed in 6/7 subjects. Bronchoalveolar lavage was performed in two subjects. No significant hemodynamic compromise was observed during any of the procedures. Significant oxygen desaturation and rising EtCO_2_ were observed in one case (patient 4). Both derangements resolved with withdrawal of the bronchoscope and recruitment. No additional complications were documented. Figure 1 illustrates evolution of the PaO_2_:FIO_2_ ratio over time for each subject. Six subjects had antibiotics modified based on FB-obtained cultures. Consistent with previous data [3], 4/7 subjects survived 30 days following discharge from the ICU.

**Table 1:** Individual patient parameters, flexible bronchoscopy performance, and outcomes (*n* = 7)

Variable	Patient 1	Patient 2	Patient 3	Patient 4	Patient 5	Patient 6	Patient 7
Age (years)	63	18	44	79	53	23	61
Sex	Female	Female	Male	Male	Male	Female	Male
Ethnicity	Black	Caucasian	Caucasian	Asian	Caucasian	Black	Caucasian
Etiology of ARDS	MRSA sepsis	Massive pulmonary embolism	Fulminant hepatic failure, *Klebsiella* sepsis	Pneumonia	Massive aspiration	Massive aspiration	Pneumonia
Total ICU LOS (days)/day of FB	27/9	30/13	97/32	35/29	9/2	49/11	16/1
Prone-positioning protocol (total hours)	28	18	16	236	20	133	18
30-day survival post ICU discharge	No	Yes	Yes	No	Yes	Yes	No
Ventilator-related parameters at FB^a^							
Mode	PRVC	PC/AC	VC/AC	PRVC	VC/AC	PC/AC	PC/AC
Peak pressure (cmH_2_O)	32	29	24	37	30	20	32
Plateau pressure (cmH_2_O)	27	NA	NA	30	26	NA	27
PEEP (cmH_2_O)	11	12	15	8	10	10	14
FIO_2_ (%)	100	100	100	100	100	100	100
FB-related data							
Δ-diameter ETT to bronchoscope (mm)	1.7	2.0	1.7	2.0	2.0	4.0	3.1
Therapeutic aspiration	Yes	Yes	Yes	No	Yes	Yes	Yes
Bronchial washings / BAL	Yes	Yes	Yes	Yes	Yes	Yes	Yes
Monitoring data							
MAP							
Baseline^a^	69	67	87	68	72	67	80
Trough during FB	69	64	72	66	71	67	68
SpO_2_							
Baseline^a^	94	98	97	100	100	100	100
Trough during FB	94	92	97	87	99	99	100
EtCO_2_							
Baseline^a^	48	30	NA	43	NA	NA	NA
Trough during FB	49	30	NA	51	NA	NA	NA
Change in antibiotic regimen based on culture results	De-escalation	De-escalation	No	Additional coverage	De-escalation	De-escalation	De-escalation

*ARDS* adult respiratory distress syndrome, *ICU* intensive care unit, *LOS* length of stay, *FB* flexible bronchoscopy, *MRSA* methicillin-resistant *Staphylococcus aureus*, *PRVC* pressure-regulated volume control, *PC/AC* pressure-cycled assist-controlled, *VC/AC* volume-cycled assist-controlled, *PEEP* positive end-expiratory pressure, *ETT* endotracheal tube, *BAL* bronchoalveolar lavage, *MAP* mean arterial pressure as measured with an arterial line, *NA* not available, *SpO*_*2*_ oxygen saturation as measured with pulse oximetry, *EtCO*_*2*_ end-tidal carbon dioxide,*FIO*_*2*_ fractional concentration of inspired oxygen

^a^As documented prior to first bronchoscope insertion

Although PP is lung-protective, it may result in mobilization of secretions into the airways, impairing oxygenation and providing nidus for infection [4]. Despite documented risks [5], FB may be beneficial in this situation.

Several limitations need to be addressed when interpreting our data. This is a retrospective analysis. Although physiologic monitoring was automatically captured, ventilator data were not and ventilator output during FB could not be accurately analyzed. Additionally, EtCO_2_ was not measured in all cases during FB. Finally, PP was shown to reduce mortality in patients with moderate to severe ARDS, however, our study subjects’ oxygenation had started to improve by the time FB was performed (Fig. 1, T1). This likely reflects reluctance to perform FB in subjects with severe hypoxemia due to excessive risks.

Our report demonstrates the feasibility of FB performed in brief increments in carefully monitored patients with ARDS ventilated in PP. Further studies are needed to better delineate optimal ventilator management during FB in PP.

## Abbreviations

ARDS: Acute respiratory distress syndrome
EtCO_2_: end-tidal carbon dioxide
FB: Flexible bronchoscopy
FIO_2_: Fraction of inspired oxygen
ICU: Intensive care unit
MV: Mechanical ventilation
PaO_2_: Partial pressure of arterial oxygen
PP: Prone position
